# Precision nutrition testing is the future of personalized care: evidence and coverage policies for equitable access

**DOI:** 10.1093/haschl/qxag247

**Published:** 2026-09-07

**Authors:** Danea M Horn, Hilary Seligman, Kathryn A Phillips

**Affiliations:** Center for Translational and Policy Research on Precision Medicine (TRANSPERS) Department of Clinical Pharmacy, University of California, San Francisco, CA 94143, United States; Philip R. Lee Institute for Health Policy Studies, University of California, San Francisco, CA 94143, United States; Philip R. Lee Institute for Health Policy Studies, University of California, San Francisco, CA 94143, United States; School of Medicine, University of California, San Francisco, CA 94143, United States; Center for Translational and Policy Research on Precision Medicine (TRANSPERS) Department of Clinical Pharmacy, University of California, San Francisco, CA 94143, United States; Philip R. Lee Institute for Health Policy Studies, University of California, San Francisco, CA 94143, United States

**Keywords:** personalized nutrition, precision nutrition, genetic nutrition testing, advanced metabolic testing, diet, insurance coverage, insurance reimbursement, health policy

## Abstract

The one-size-fits-all approach to dietary and metabolic interventions often fails to improve population health outcomes. This problem has been made more urgent by the rise of GLP-1 receptor agonist medications for obesity, which are high-cost but have variable patient outcomes. Precision nutrition (PN) testing has the potential to address this gap by using biomarkers, such as genetics and metabolic profiles, to predict individual responses to foods and drug therapies. Although some PN tests are on the market, most are not FDA-approved or covered by insurers, requiring out-of-pocket payment and creating access disparities. In this Commentary, we draw on experience from precision medicine to argue that payers, developers, regulators, and professional societies must begin building the evidence and coverage infrastructure for PN testing now. Payer coverage will require demonstrated clinical utility and defined clinical pathways that guide who should be tested and how results should change care. The policy question is not whether current tests should be covered; they should not be broadly covered without evidence, but whether the health system is preparing for equitable implementation once validated tests emerge. Building evidence and coverage infrastructure proactively will help ensure that future PN testing innovations improve population health equitably rather than widen existing disparities.

Key Points

1. Precision nutrition (PN) testing is in its infancy, but validated tests that can target dietary and metabolic interventions, including identifying which patients will respond to GLP-1 medications, are on the horizon.
2. Insurance coverage is essential for equitable access to PN testing and requires demonstrated clinical utility and well-defined clinical pathways.
3. Payers, developers, and regulators must develop the evidence base and build coverage infrastructure now to prevent a repeat of precision medicine's history of delayed and unequal access.

## Introduction

The one-size-fits-all approach to dietary guidelines and nutritional and metabolic interventions often fails to improve health outcomes across populations.^1^ These issues became more urgent with the emergence of high-cost, widely prescribed GLP-1 receptor agonist medications for obesity treatment. However, GLP-1s do not work for everyone, with considerable side effects and an 18% non-response rate.^2^ Without tests to identify patients most likely to benefit, payers have responded to escalating costs with restrictive coverage policies, creating access disparities that disadvantage patients who cannot pay out of pocket.

Precision nutrition (PN) testing is an emerging field that uses biomarkers, such as genetics, microbiome composition, and comprehensive metabolic profiles, to predict how a person will respond to specific foods and drug therapies for disease prevention and optimal health.^1^ Recognizing this potential, the National Institutes of Health invested $170 million in the Nutrition for Precision Health Initiative to develop algorithms that predict individual responses to dietary interventions. Establishing PN-specific evidentiary thresholds for clinical utility and building the evidence and coverage infrastructure now, even before validated tests are ready for clinical use, is central to ensuring that health benefits are realized equitably.

Although there are some PN tests now on the market, many are not FDA-approved or covered by insurers and are marketed directly to consumers, requiring out-of-pocket payments that create access disparities.^3^ Our decades of work in precision medicine have demonstrated that early consideration of payer coverage is essential for evidence development and timely, equitable access.^4^ In this Commentary, we examine what needs to happen now to ensure that PN testing reaches the patients who can benefit the most, with particular focus on the evidentiary requirements and payer coverage considerations.

## Precision nutrition testing is increasingly necessary

PN testing is in its infancy, similar to precision medicine 2 decades ago, when the field showed promise but lacked the standardized evidence infrastructure needed for widespread clinical adoption. However, there is increasing evidence that PN testing could be clinically useful, eg, recent research has identified specific genetic variants that predict whether a patient will respond to GLP-1 medications and which side effects they may experience before treatment begins.^5^ However, for emerging PN tests to achieve success in targeting dietary and metabolic interventions for adult populations, as we have seen with precision medicine tests such as for cancer therapies, they must overcome critical coverage barriers: lack of clinical utility evidence and undefined clinical pathways.^4^

## Clinical utility evidence must be established before coverage

The primary evidence supporting payer coverage policies is clinical utility, meaning that a test changes clinical decision-making in ways that improve patient outcomes. Most current studies of PN tests show associations between certain genes or other biomarkers and health outcomes but have not yet shown that acting on those findings improves patient care. Without demonstrating that test results lead to different therapeutic interventions, better dietary adherence, or reduced disease incidence compared to standard care, payers are unlikely to cover PN tests.^4^ The threshold for payers to cover tests will vary based on the specific test and clinical context. However, prior research suggests that although clinical utility is the driving factor, other factors such as FDA approval, testing costs relative to benefits, and patient/physician demand can contribute to positive coverage decisions. What constitutes sufficient clinical utility evidence for PN tests specifically (ie, which actionable interventions and outcome measures will satisfy payers) has not been established and will require input from professional societies, clinicians, payers, and regulators.

### Example: the cautionary tale of genetic testing for hereditary breast cancer risk

Precision medicine offers a clear lesson on the consequences of delayed payer coverage for tests that inform treatment. BRCA1/2 genetic testing for hereditary breast cancer risk demonstrated strong clinical utility, as individuals who tested positive could take action to reduce their cancer risk significantly.^6^ Yet for years, coverage was inconsistent, and a large share of patients paid out of pocket, limiting access to those who could afford it.^7^ PN testing risks repeating these access disparities. We are not advocating for coverage without appropriate evidence, but rather that the time to build coverage infrastructure is before validated tests arrive, not after.

### Example: clinical utility and nutrition testing using genetic profiles

There is a growing market for PN testing, based on individual genetic profiles, offered directly to "healthy" individuals seeking personalized dietary guidance (“nutrigenomics”). However, concerns have been raised about the lack of regulation and scientific validity of some tests, with marketing claims that often exceed current evidence.^3^ Studies have not demonstrated that this testing leads to better dietary adherence, greater weight loss, or reduced disease incidence compared to standard practice, and thus, testing has not been integrated into clinical practice guidelines.^8^ PN testing using genetic profiles thus provides an example of how establishing clinical utility evidence through prospective trials or real-world evidence is necessary to support coverage. At the same time, high patient demand can increase costs of care without demonstrated benefits, and thus, insurers will not cover PN tests until evidence is established.

## Coverage requires defined clinical pathways

Beyond clinical utility evidence, payer coverage for PN tests will need well-defined clinical pathways that guide pre-testing decisions (who should be tested) and post-testing actions (how results should change care). These pathways identify who will benefit and who should be excluded from testing and treatment. Coverage matters throughout this pathway, as payers must decide if reimbursement is limited to the diagnostic test itself or extends to the clinical workup needed to determine PN testing eligibility and the downstream treatment(s) and monitoring needed to act on results. While all PN tests require clinical pathways for coverage, we use testing for GLP-1s as an example, given that new approaches to testing are on the horizon.

### Pre-testing pathway

Pre-testing pathways determine which patient populations should receive testing based on clinical indicators, family history, or risk factors. For GLP-1s, this pathway would identify who should receive testing to predict whether they are likely to respond to treatment before a prescription is written. Current prescribing criteria target adults with a BMI of 30 or above, or a BMI of 27 or above with a weight-related comorbidity (eg, diabetes or heart disease), but consensus has not been reached across organizations.^9,10^ The key question will be whether the same prescribing criteria should guide who receives a GLP-1 screening test, when available, or whether testing warrants its own eligibility standards. Without clear pre-testing criteria, the potential for widespread use and misuse of testing is considerable, which could increase health care costs if testing is applied broadly rather than targeted to patients most likely to benefit. Conversely, well-targeted criteria could avoid the costs associated with non-response and early discontinuation, which affect a substantial share of current GLP-1 users.

### Post-testing pathway

Coverage is also more likely when abnormal test results trigger clear, evidence-based treatment pathways. In the case of GLP-1s, a test would predict if the patient would benefit from a prescription. However, post-testing pathways will need to extend beyond initiation of GLP-1s. The WHO recommends long-term, continuous GLP-1 use combined with intensive behavioral therapy;^9^ yet, evidence on how to manage treatment over time remains limited, and up to 50% of patients discontinue within 12 months, often leading to weight regain.^10^ Post-testing pathways must therefore extend beyond the initial prescription to include ongoing monitoring, side effect management, and guidance on when to adjust or stop treatment.

## Conclusion

PN testing is increasingly important for identifying which patients will respond to treatments before prescribing, thereby saving costs, improving outcomes, and reducing adverse effects.^5^ The policy question is not whether current tests should be covered; they should not be broadly covered without evidence, but whether the health system is preparing for equitable implementation once validated tests emerge. The history of precision medicine has already demonstrated the consequences of delayed coverage planning, including fragmented access, out-of-pocket burdens, and widening disparities. Insurance coverage alone will not ensure access, given large numbers of uninsured or underinsured individuals, but it is a starting point.

Developers, professional societies, government agencies (eg, FDA and CMS), and payers must begin now to build the evidence and coverage infrastructure needed to establish clinical utility and define clear pre- and post-testing pathways to guide clinical implementation of PN tests. One means of doing so is coverage with evidence development policies that tie reimbursement to ongoing evidence generation, which could help steward PN testing into clinical care. Building coverage infrastructure before validated tests enter routine use will help ensure that future innovations in PN, including tests that could target high-cost therapies such as GLP-1 receptor agonists, improve population health equitably rather than widen existing disparities.

## Supplementary Material

qxag247_Supplementary_Data

## References

[1] NIH . *2020–2030 Strategic Plan for NIH Nutrition Research A Report of the NIH Nutrition Research Task Force*. National Institutes of Health, United States Department of Health and Human Services; 2020. Accessed September 5, 2025. https://dpcpsi.nih.gov/onr/strategic-plan

[2] Squire P, Naude J, Zentner A, Bittman J, Khan N. Factors associated with weight loss response to GLP-1 analogues for obesity treatment: a retrospective cohort analysis. BMJ Open. 2025;15(1):e089477. 10.1136/bmjopen-2024-089477 PMC11751938 39819958

[3] Rogus S, Lurie P. Personalized nutrition: aligning science, regulation, and marketing. Health Aff Sch. 2024;2(9):qxae107. 10.1093/haschl/qxae107 PMC11382137 39253562

[4] Phillips KA . Assessing the value and implications of personalized/precision medicine and the “lessons learned” for emerging technologies: an introduction. Value Health. 2017;20(1):30–31. 10.1016/j.jval.2016.09.2405 28212965

[5] Su QJ, Ashenhurst JR, Xu W, et al Genetic predictors of GLP1 receptor agonist weight loss and side effects. Nature. 2026;653(8115):770–775. 10.1038/s41586-026-10330-z PMC13190316 41951734

[6] Knerr S, Bowles EJA, Leppig KA, Buist DSM, Gao H, Wernli KJ. Trends in BRCA test utilization in an integrated health system, 2005–2015. J Natl Cancer Inst. 2019;111(8):795–802. 10.1093/jnci/djz008 PMC6695306 30753636

[7] Cragun D, Weidner A, Lewis C, et al Racial disparities in BRCA testing and cancer risk management across a population-based sample of young breast cancer survivors. Cancer. 2017;123(13):2497–2505. 10.1002/cncr.30621 PMC5474124 28182268

[8] Braakhuis A, Monnard CR, Ellis A, Rozga M. Consensus report of the academy of nutrition and dietetics: incorporating genetic testing into nutrition care. J Acad Nutr Diet. 2021;121(3):545–552. 10.1016/j.jand.2020.04.002 32624395

[9] Celletti F, Farrar J, De Regil L. World health organization guideline on the use and indications of glucagon-like peptide-1 therapies for the treatment of obesity in adults. JAMA. 2026;335(5):434–438. 10.1001/jama.2025.24288 41324410

[10] Thomsen RW, Mailhac A, Løhde JB, Pottegård A. Real-world evidence on the utilization, clinical and comparative effectiveness, and adverse effects of newer GLP-1RA-based weight-loss therapies. Diabetes Obes Metab. 2025;27 Suppl 2(Suppl 2):66–88. 10.1111/dom.16364 PMC12000858 40196933

